# Rivastigmine Lowers Aβ and Increases sAPPα Levels, Which Parallel Elevated Synaptic Markers and Metabolic Activity in Degenerating Primary Rat Neurons

**DOI:** 10.1371/journal.pone.0021954

**Published:** 2011-07-22

**Authors:** Jason A. Bailey, Balmiki Ray, Nigel H. Greig, Debomoy K. Lahiri

**Affiliations:** 1 Department of Psychiatry, Institute of Psychiatric Research, Indiana University School of Medicine, Indianapolis, Indiana, United States of America; 2 Medical and Molecular Genetics, Indiana University School of Medicine, Indianapolis, Indiana, United States of America; 3 Laboratory of Neuroscience, Intramural Research Program, National Institute of Aging, National Institutes of Health, Baltimore Maryland, United States of America; National Institute on Aging Intramural Research Program, United States of America

## Abstract

Overproduction of amyloid-β (Aβ) protein in the brain has been hypothesized as the primary toxic insult that, via numerous mechanisms, produces cognitive deficits in Alzheimer's disease (AD). Cholinesterase inhibition is a primary strategy for treatment of AD, and specific compounds of this class have previously been demonstrated to influence Aβ precursor protein (APP) processing and Aβ production. However, little information is available on the effects of rivastigmine, a dual acetylcholinesterase and butyrylcholinesterase inhibitor, on APP processing. As this drug is currently used to treat AD, characterization of its various activities is important to optimize its clinical utility. We have previously shown that rivastigmine can preserve or enhance neuronal and synaptic terminal markers in degenerating primary embryonic cerebrocortical cultures. Given previous reports on the effects of APP and Aβ on synapses, regulation of APP processing represents a plausible mechanism for the synaptic effects of rivastigmine. To test this hypothesis, we treated degenerating primary cultures with rivastigmine and measured secreted APP (sAPP) and Aβ. Rivastigmine treatment increased metabolic activity in these cultured cells, and elevated APP secretion. Analysis of the two major forms of APP secreted by these cultures, attributed to neurons or glia based on molecular weight showed that rivastigmine treatment significantly increased neuronal relative to glial secreted APP. Furthermore, rivastigmine treatment increased α-secretase cleaved sAPPα and decreased Aβ secretion, suggesting a therapeutic mechanism wherein rivastigmine alters the relative activities of the secretase pathways. Assessment of sAPP levels in rodent CSF following once daily rivastigmine administration for 21 days confirmed that elevated levels of APP in cell culture translated in vivo. Taken together, rivastigmine treatment enhances neuronal sAPP and shifts APP processing toward the α-secretase pathway in degenerating neuronal cultures, which mirrors the trend of synaptic proteins, and metabolic activity.

## Introduction

Alzheimer's disease (AD) is the most common cause of dementia in the elderly, afflicting approximately 5.4 million people in the United States [1] with steady increases projected to at least mid-century as the current population gradually ages [2]. AD is a mutifactorial disorder with an unclear etiology. Some of the potential causative or contributing factors implicated in AD include genetic and epigenetic factors [3], [4], diet and physical activity [5], education [6], heavy metal exposure [7], and other gene-environment interactions [8]. Psychological attributes of the individual, such as a subjective sense of a purposeful life, may also correlate with risk of developing AD [9].

AD is diagnosed *post mortem* based on the presence in brain of amyloid plaques, comprised mostly of amyloid-β (Aβ) peptides, and neurofibrillary tangles (NFTs) of hyperphosphorylated microtubule associated protein tau (MAPT). Aβ, together with other proteolytic fragments of dysregulated amyloid precursor protein (APP) processing, have been implicated as major mediators of neurotoxicity in AD [10] and are known to induce toxic effects through a wide variety of mechanisms.

One important therapeutic goal in AD treatment is to restrict the progression and/or delay the onset of the disease. Currently, the cholinesterase inhibitors (ChEI) tacrine, donepezil, rivastigmine, galantamine and a partial NMDA receptor antagonist memantine are the only drugs approved by the FDA for treatment of AD [11], [12]. The main aim of ChEI treatment is to inhibit enzymatic degradation of the neurotransmitter acetylcholine, resulting in an increased amount of acetylcholine in the synaptic terminals [13], [14]. Memantine is thought to protect neurons from glutamate induced excitatory damage [15], and may also modulate Aβ production [16]. However, the aforementioned FDA approved drugs do not adequately restrict or reverse the progression of AD. Therefore, several different approaches, such as regulating levels of Aβ precursor protein (APP) by novel microRNA mediated mechanisms, are under investigation [17].

APP is a type I transmembrane protein that undergoes proteolytic processing by secretase enzymes to generate soluble fragments [18]. Through alternative splicing, APP is generated in several different forms in various tissues throughout the body. In the brain these range from 695 to 770 amino acids, based on alternative splicing of the Kunitz protease inhibitor domain (KPI) and MRC OX-2 antigen (OX-2) domains [19]. Previous work has suggested that the longer KPI-containing APP is produced mainly by gilal cells, while the shorter non-KPI containing splice variant is primarily or neuronal origin [20]. Furthermore, there is evidence that a shift in APP isoforms favoring production of KPI-containing APP may contribute to amyloidogenesis [21]. Sequential cleavage of APP by β-secretase (BACE1) and the γ-secretase complex produces sAPPβ and Aβ peptide, the major component of amyloid plaques found in AD [22]. Non-amyloidogenic cleavage by α-secretase and γ-secretase releases the carboxyl-truncated secreted sAPPα and non-amyloidogenic 3 kDa peptide (p3), instead of intact 4 kDa Aβ [22]. Notably, the predominant sAPPα species has been shown to exhibit a wide array of neurotrophic activities [23], [24], which are important for neuron development and survival. We postulate that elevated levels of sAPPα could participate in cellular metabolic activity, enhancing neuron survival under pathological conditions such as AD.

Contrary to sAPPα, higher levels of Aβ are known to be toxic. It has been reported that ganglioside-bound Aβ initiates Aβ aggregation by acting as a seed [25], which could lead to aggregation states with enhanced toxicity. Aβ derived diffusible ligands (ADDLs) are soluble multimers of Aβ that are thought to be the predominant source of Aβ-mediated toxicity in the AD brain [26]. Another important mechanism of Aβ toxicity that is particularly pertinent to memory impairment is a reduction in functional synapses [27]. Synapse loss is not part of the definitive criteria required for AD diagnosis, however, it has been consistently found that synapse density may correlate more closely with the clinical manifestations of AD than plaque or NFT density [28], and occurs early in the course of AD [29]. Recent evidence suggests that secreted APP (sAPP) is protective against neuronal apoptosis [30], and thus may mitigate some of the toxic events that occur in the AD brain.

We have recently demonstrated that rivastigmine (Exelon^tm^, Novartis), a dual acetyl- and butyrylcholinesterase inhibitor, can preserve neuronal morphology as well as pre-synaptic protein markers in our degenerating primary neuronal culture model [31]. Rivastigmine is a novel intermediate-acting reversible non-competitive carbamate dual acetyl- and butyryl-cholinesterase inhibitor which is currently used for the treatment of AD [32], however, its mode of action is incompletely understood. Others have shown that sAPP can prevent neurite retraction in response to proteasome stress [33]. Herein, we test the hypothesis that the effects of rivastigmine on synaptic protein levels, neuronal morphology, and metabolic activities may be mediated, in part, by modulation of APP processing and metabolic activity of the cells. Rivastigmine is an established therapeutic agent used to treat AD, and a more complete understanding of its actions on neuronal cells can be expected to both optimize its clinical potential and aid future drug development efforts by providing new drug targets and experimental endpoints. To this end, we treated degenerating primary fetal rat cortical cell cultures with rivastigmine and measured secretion of total APP, sAPPα, and Aβ peptide and compared these to levels of pre- and post-synaptic markers, and metabolic activity. Rivastigmine treatment induced an elevation in both metabolic activity and APP secretion, and differentially impacted two isoforms of sAPP, which are distinguishable by molecular weight. In our cultures, low molecular weight sAPP corresponded closely to neuronal viability, and high molecular weight sAPP corresponded with glial proliferation. Our results suggest that within the mixed culture system used, neurons are the primary source of sAPP, and rivastigmine's actions are mediated principally through neuronal, rather than glial, targets. Importantly, rivastigmine was found to increase the neurotrophic sAPPα and decrease Aβ secretion, suggesting a mechanism for the previously observed neuropreservation effects. These changes in APP processing were accompanied by increased pre- and post-synaptic markers, suggesting a possible role for APP secretion in the effects of rivastigmine. These findings were extended to an *in vivo* model in which increased sAPP levels were found in the cerebrospinal fluid (CSF) of rats treated with rivastigmine daily for three weeks. Taken together, our results suggest that rivastigmine treatment enhances neuronal sAPP in degenerating neuronal cultures. This effect mirrors the trend of neuronal and synaptic proteins, and cellular metabolic activity. Further, rivastigmine's effect was translated to *in vivo* studies in a wild-type animal strain. Based on the results from our unique primary degenerating culture model and separate *in vivo* studies, we suggest that rivastigmine's novel positive effect on neuronal viability would further help to optimize this drug for the treatment of AD.

## Materials and Methods

### Ethics Statement

All procedures involving animals were approved by the Institutional Animal Care and Use Committee of Indiana University, and conform to NIH guidelines (study #2872).

### Primary embryonic rat cerebrocortical cultures

Primary embryonic rat cortical cultures were generated using previously published procedures [34], with small modifications as described previously [31]. Cerebrocortical tissue from embryonic day 16 rat pups was collected and dissociated by trituration with a fire-polished Pasteur pipette. Cells were seeded into 12-well tissue culture treated, poly-D-lysine (Sigma-Aldrich, St. Louis, MO) coated, polystyrene multiwell plates (Corning, Lowell, MA). Cells were maintained in Neurobasal medium supplemented with B27 serum replacement, antibiotic cocktail, 5 ng/ml basic fibroblast growth factor, 0.5 mM L-glutamine (Invitrogen, Carlsbad, CA), and 1× Normocin (InVivoGen, San Diego, CA). Media were changed every fourth day until day 14 when rivastigmine was added during a final media change. Cells were treated until day 16 when media samples were collected and cells were removed from the plate by scraping with a rubber-tipped spatula in Dulbecco's phosphate buffered saline (D-PBS; Invitrogen). Cells were pelleted by centrifugation and lysed by sonication in lysis buffer containing 50 mM Tris-HCl (pH 7.0–7.5), 150 mM NaCl, 5 mM EDTA, 0.5% Triton X-100, 0.5% sodium deoxycholate (all from Sigma-Aldrich) and 1× Complete Mini protease inhibitor cocktail (Roche, Indianapolis, IN).

### Characterization of an in vitro neurodegeneration model

Previous experience with cells cultured under the above conditions suggests that the neurons in this mixed culture degenerate with time while glia proliferate. This presents a possible model for testing various drugs for potential neuropreservative effects. To more clearly define this model, samples were taken at 12 days and 20 days *in vitro* and cell lysates were examined by Western blot for levels of neuron-specific enolase (NSE), glial fibrillary acidic protein (GFAP), and the conditioned media samples were examined for levels of various sAPP isoforms. Results from the present manuscript should be considered in the context of the effect of rivastigmine in the degenerating primary cerebrocortical cultures and not in a static cell culture model.

### Rivastigmine extraction from capsules

Rivastigmine was provided as a gift by Dr. Martin Farlow as 1.5 mg pharmaceutical capsules. The contents of the capsules were dissolved in sterile water and disrupted by sonification, then clarified by centrifugation to yield a 5 mM stock solution. Other contents of the capsules included hydroxypropyl methylcellulose, magnesium stearate, microcrystalline cellulose, and silicon dioxide, and are generally considered as pharmaceutically inert [35]. The concentration of this rivastigmine stock was subsequently confirmed by comparison to an analytical standard (provided by Novartis) by UV-Vis spectrometry using a Nanodrop spectrophotometer (Thermo Scientific, Waltham, MA).

### Drug treatment conditions

In the cell culture experiments, rivastigmine treatments were performed by adding the 5 mM stock solution of capsule-extracted drug in to the Neurobasal based growth medium described above at the concentrations indicated in the figures. In all experiments, untreated or “vehicle treated” cells were included for comparison to the rivastigmine-treated cells.

### ATP-based metabolic activity assessment

Relative ATP concentrations were measured using the luciferase based Cell Titer-Glo kit (CTG; Promega, Madison, WI), as described previously [16]. After treatment, cells were rinsed once in cold PBS and then collected by scraping with a rubber-tipped spatula. Thirty microliter aliquots of these cell suspensions in D-PBS were transferred to an opaque white 96-well plate (Corning, Lowell, MA) and 30 ul of the CTG assay solution was added and the plate was placed on an orbital shaker for 10 minutes to induce lysis of the cells. The luminescent signal was quantified using a Glowmax luminometer (Promega).

### Lactate dehydrogenase-based toxicity assay

Cells were monitored for toxic effects of rivastigmine treatment using a commercially available lactate dehydrogenase (LDH) based kit (Sigma-Aldrich). The LDH enzyme is a cytosolic component of the glycolytic pathyway, and leakage from the cytoplasm into the cell culture medium is an indication of membrane permeability, which results from cellular toxicity. At the conclusion of an experiment, 30 µl aliquots of the conditioned medium were assayed from each well. LDH concentration in the media samples was calculated by comparison to standards of known LDH content (Roche).

### Western immunoblotting

Relative APP protein levels were examined in media samples of rivastigmine treated cells by standard Western blot with immunodetection techniques, as described previously [16]. Briefly, media samples were resolved under SDS-induced denaturing conditions on 15-lane, 10% polyacrylamide gel using the Mini Protean II system for larger sample volumes, or on 10% polyacrylamide 26-lane Criterion-XT gels for larger sample numbers (Bio-Rad, Hercules, CA) then transferred to 0.2 µm poresize PVDF. Blots were probed with monoclonal mouse-anti-APP (22C11; Millipore, Billerica, MA), polyclonal rabbit-anti-KPI (KPI; Abcam, Cambridge, MA) to detect long splice variant APP KPI, and monoclonal mouse-anti-β-actin (Sigma-Aldrich) as an internal control. The blots were then probed with the appropriate horseradish peroxidase-conjugated secondary antibody (Pierce, Rockford, IL) and detected by ECL techniques (GE Healthcare, Piscataway, NJ).

To detect proteins found in the lysates of cultured cells, protein concentrations of the lysates were determined by the Bradford technique (BioRad), and 10 µg of each lysate protein was subjected to electrophoresis and Western blotting under conditions identical to the media samples. Blots of the lysates were probed with mouse-anti-SNAP-25 (Millipore), mouse-anti-PSD-95 (Antibodies Incorporated, Davis, CA), mouse-anti-syntaxin-4 (BD Transduction Labs, Franklin Lakes, NJ), mouse anti-β-actin (Sigma-Aldrich), and the 22C11 antibody as above, followed by the appropriate secondary antibody and ECL detection.

### Enzyme linked immunosorbent assays (ELISAs)

Measurement of α-secretase cleaved secreted APP (sAPPα) in the conditioned medium (CM) was undertaken by by a sensitive and specific ELISA (IBL, Gumma, Japan). The assay was performed per manufacturer's protocol. Briefly, 30 µl of CM samples were loaded in capture antibody (anti rodent APP; clone 597) coated wells of the ELISA plate and incubated overnight at 4°C. After several washes the wells were incubated with detection antibody (HRP-conjugated anti rodent. APP; clone 18) for 30 min at 4°C. After additional washes, chromogen solution was added into each well and kept at room temperature for 30 min. The reaction was stopped by adding 1N H_2_SO_4_ and colorimetric signals were recorded (450 nm) using a Model 550 microplate reader (BioRad). A standard curve with known amounts of recombinant rodent sAPPα was also generated. sAPPα values were normalized by the total protein content of the CM samples and plotted as pg/mg of protein present in CM samples.

Measurement of Aβ_x-40_ in the CM samples was carried out by using a sensitive chemiluminescence ELISA (Covance , Princeton, NJ) as described previously [36]. Chemiluminescence signals were normalized by the total protein content of CM samples to obtain pg of Aβ_x-40_ present in per mg of protein of CM samples. This Aβ ELISA kit detects full length sequence of Aβ_1–40_ and also peptides generated by N-terminal cleavage of APP, such as Aβ_3–40_, Aβ_11–40_ etc.

### Immunocytochemistry

Cultures were maintained until day 12 or day 20 and then rinsed once in Dulbecco's phosphate buffered saline (DPBS; Invitrogen, Carlsbad, CA) and fixed in the cell culture plate with 4% paraformaldehyde. Cells were then permeabilized with 0.5% Triton X-100 (Sigma) and non-specific binding was blocked with 10% horse serum (Atlanta Biologicals, Lawerenceville, GA). Mouse anti-MAP2 (Abcam) followed by a biotinylated donkey-anti-mouse secondary antibody and FITC-conjugated streptavidin (Jackson IR, West Grove, PA) or rabbit anti-GFAP (Sigma-Aldrich) followed by Cy3 conjugated donkey-anti-rabbit secondary antibody (Jackson IR). Cells were visualized using a Leica DM-IL inverted microscope (Wetzlar, Germany) fitted with a SPOT RT-SE camera (Diagnostic Instruments, Sterling Heights, MI). These time points were selected because our previous observations of these cultures indicated that neuronal morphology peaked around day 12, and by day 20, the neuronal population subsided and glia had become prominent (unpublished observations).

### Study of rivastigmine effects on sAPP in vivo

Male Fischer-344 rats, weighing approximately 200 g (Harlan, Indianapolis, IN), were separated into groups of 8–12 animals and treated for 21 days with either rivastigmine (0.75 mg/kg i.p., prepared in 1 ml/kg volume isotonic saline) or vehicle (isotonic saline only). In addition, a separate group was administered phenserine tartrate (2.5 mg/kg i.p., in 1 ml/kg isotonic saline) as a positive control, as this compound has been consistently reported to lower the rate of APP synthesis and APP levels in the brain [37]. Thereafter, rats were killed within 90 to 120 min of their final dosing and CSF was immediately obtained from the cisterna magna, and frozen at −70°C until assayed for sAPP levels.

Levels of total sAPP in CSF samples (5 µl) were assayed by Western immunoblot analysis as previously described [16] and probed with anti-APP antibody, 22C11. This recognizes all forms of APP in CSF, as well as the APP-like proteins (APLP). The former migrated as two bands (110–130 kDa), whereas the latter migrated at a slightly different rate, to (70 kDa), and were not quantitated.

### Data analysis

Western blot data (ECL films) were scanned and quantified using ImageJ software [38]. Statistical analysis was performed using one-way ANOVA with Tukey post-hoc test (SPSS software, v. 17). A p-value of <0.05 was considered statistically significant.

## Results

### Development of a neurodegenerating model of primary cerebrocortical cultures: Transition of cell types and sAPP isoforms over time

**Figure 1:**
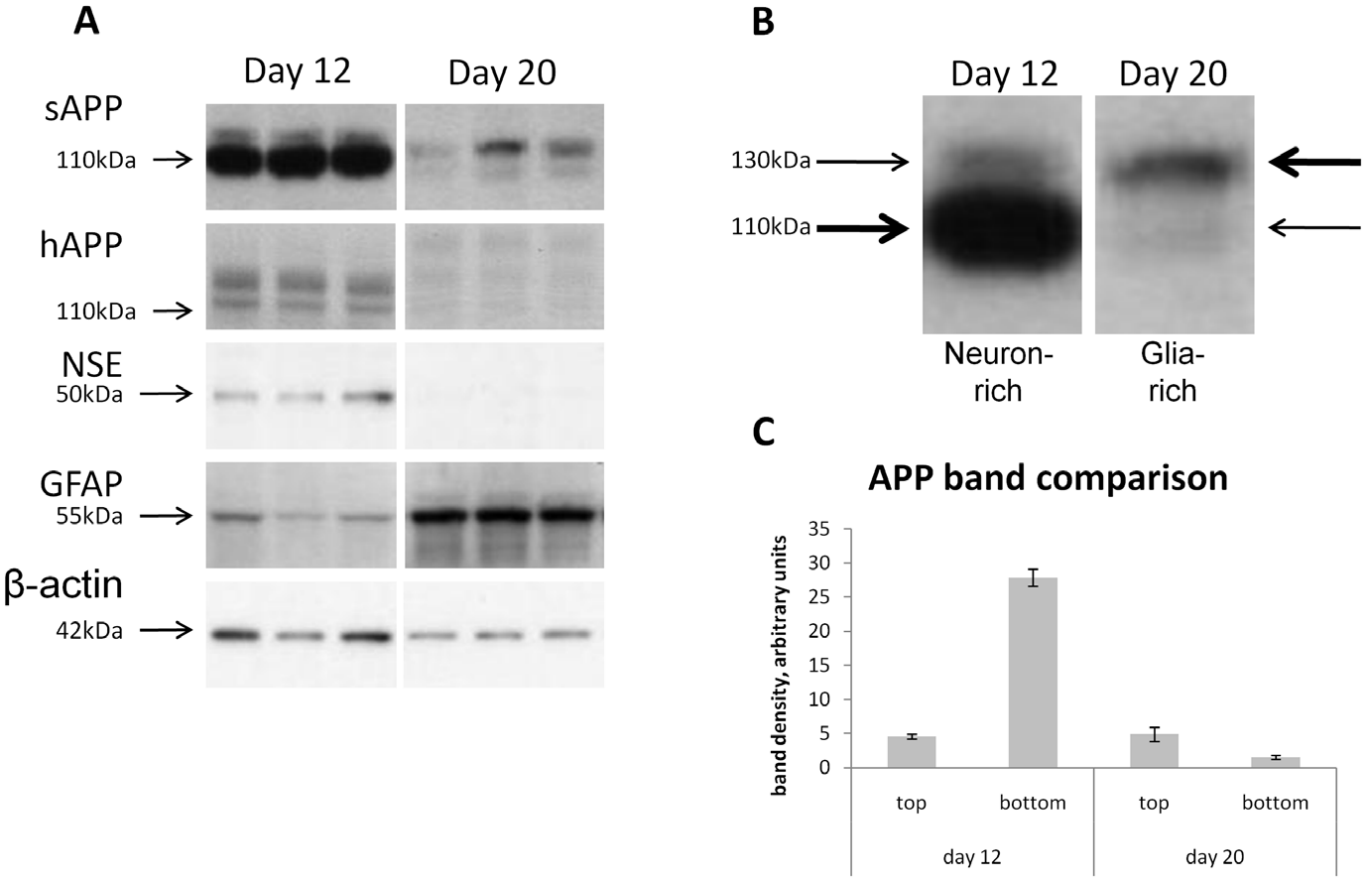
Establishment of a neurodegenerating model of primary cerebrocortical cultures: Association of different APP isoforms with glial and neuronal cell types. **A**: Cell lysates and conditioned media samples of primary cortical cultures were collected at day 12 and day 20 for Western blot analysis. At day 12 compared to day 20, levels of the neuronal marker NSE are high, and levels of the glial marker GFAP are low. This indicates that at day 12 the neuronal population is intact, whereas the glial population becomes more pronounced at day 20, and neurons have degenerated by day 20. In the sAPP blot, reduced levels of low molecular weight sAPP coincide with the loss of neurons, while the increased high molecular weight sAPP parallels the increase in glia abundance. Approximate molecular weights are indicated. Day 12 and day 20 samples were examined on the same blots with irrelevant samples cropped from the images. **B**: A detailed view of sAPP bands in single representative lanes of Western blots from neuron-rich (day 12) and glia-rich (day 20) cell cultures reveals that the lower band (∼110 kDa) is predominant over the upper band (∼130 kDa) in neuron-rich cultures This pattern is reversed in glia-rich cultures, with a predominant upper band, and very little sAPP present at the lower molecular weight. **C**: Densitometric quantification of the top vs. bottom bands from day 12 and day 20 samples is also shown. Levels of the high molecular weight top band are relatively stable with time, while levels of the low molecular weight bottom band decrease dramatically between day 12 and day 20, concurrent with neurodegeneration.

**Figure 2:**
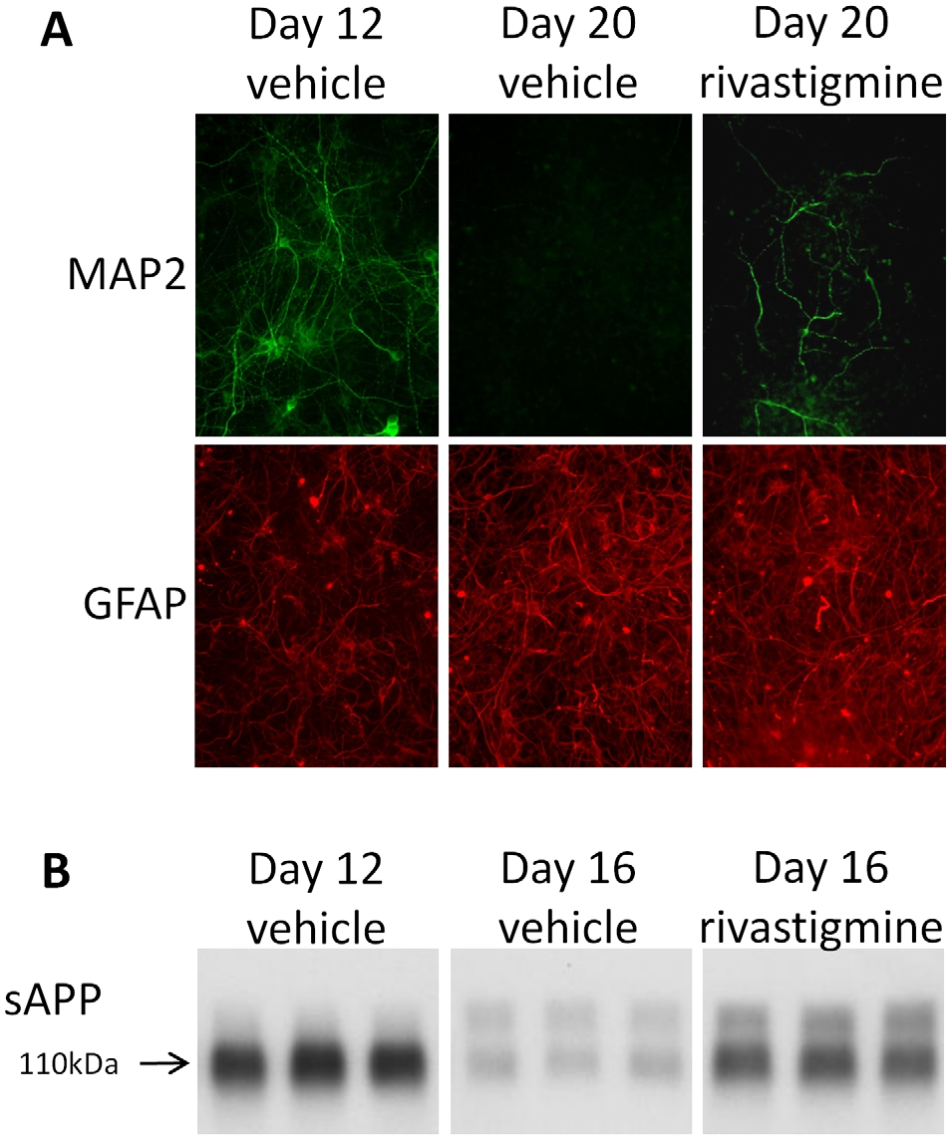
Rivastigmine preserves neuronal morphology and alters sAPP secretion. **A**: At day 12 or day 20, cells were fixed for immunocytochemistry analysis and probed with anti-MAP2 (green) and anti-GFAP (red) antibodies. Neuronal MAP2 immunoreactivity declines to almost undetectable levels between day 12 and day 20 in untreated cells, whereas neuronal morphology is preserved in rivastigmine treated cultures. Glial GFAP was observed to increase in both treated and untreated cells between day 12 and day 20. These results confirm the degeneration of neurons by day 20. All samples were examined on the same blot with irrelevant samples cropped from the images. **B**: In a separate experiment, media samples were taken before the onset of neurodegeneration at day 12 and during the degenerating phase of the cell culture at day 16. The lower molecular weight neuronal form of sAPP declined significantly in untreated cells, but was rescued by 10 µM rivastigmine treatment.

To examine the sources of sAPP in the conditioned media of these cultures, media and lysate samples from cells maintained for 12 days or 20 days *in vitro* were compared by Western blotting and immunocytochemistry techniques (figures 1 and 2). We have observed that the neuronal component of these mixed primary cultures peaked around day 12, as confirmed by the presence of NSE in the lysates (figure 1A) and robust neuronal morphology observed by MAP2 immunocytochemistry (figure 2). Simultaneously, the glial component began to increase around day 12, as observed by the presence of GFAP by Western blot and immunocytochemistry. At day 20, NSE was undetectable in the lysates and very little MAP2 was visualized by immunocytochemistry, indicating that the neuronal component is almost completely absent. At day 20, greatly increased GFAP was observed by both Western blot and immunocytochemistry. Please note that all samples comparing cell type markers at different time points were run in the same gel with irrelevant (e.g., other drug treatments) cropped out for clarity. When compared to the relative intensities of the two major sAPP bands, the low molecular weight band was very strong at day 12, and appeared to associate with the neuronal component. The high molecular weight band was more prominent at day 20 when the glial component had become prominent (figure 1B). The levels of APP holoprotein (hAPP) in the cell lysates during this time was also reduced and shifted toward higher molecular weight forms (figure 1A).

### Rivastigmine treatment increased metabolic activity and preserved neuronal viability

**Figure 3:**
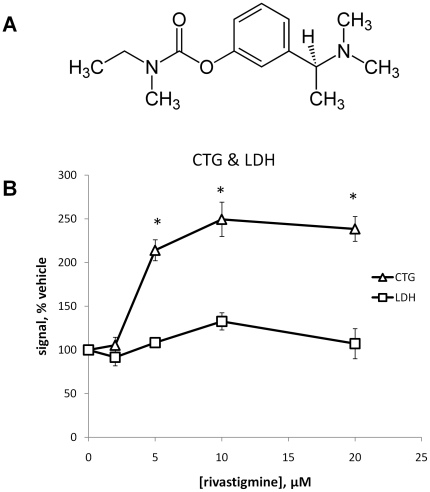
Rivastigmine enhances cell viability in neurodegenerating primary cultures. **A**: Rivastigmine is a parasympathomimetic cholinergic agent used for the treatment of mild to moderate AD. Its systematic (IUPAC) name is (S)-N-Ethyl-N-methyl-3-[1-(dimethylamino)ethyl]-phenyl carbamate. **B**: Primary cultures were treated with rivastigmine at the indicated concentrations for 4 days starting at day 12, and were then subjected to the Cell Titer-Glo (CTG) assay to assess cell viability and metabolic activity. Significant increases in ATP concentrations were observed in the lysates of cells treated with 5 µM, 10 µM and 20 µM rivastigmine (both p<0.05), while no effect was observed with 2 µM rivastigmine. The potential of drug-induced toxicity was assessed by measuring LDH release, but no significant difference from vehicle was observed, suggesting that all rivastigmine doses were well tolerated.

Cell viability and metabolic activity as measured by the CTG assay increased dose-dependently at 5 µM, 10 µM and 20 µM rivastigmine but not 2 µM (figure 3B). While this is a mixed culture and effects on glial cells cannot be ruled out completely, data presented here (figure 3a), as well as previously reported data [31], suggest that the observed increase in viability is primarily due to preservation of neurons. LDH release from the cells was not affected by any concentration of rivastigmine tested, indicating no toxicity.

### Rivastigmine treatment preserved neuronal structure

**Figure 4:**
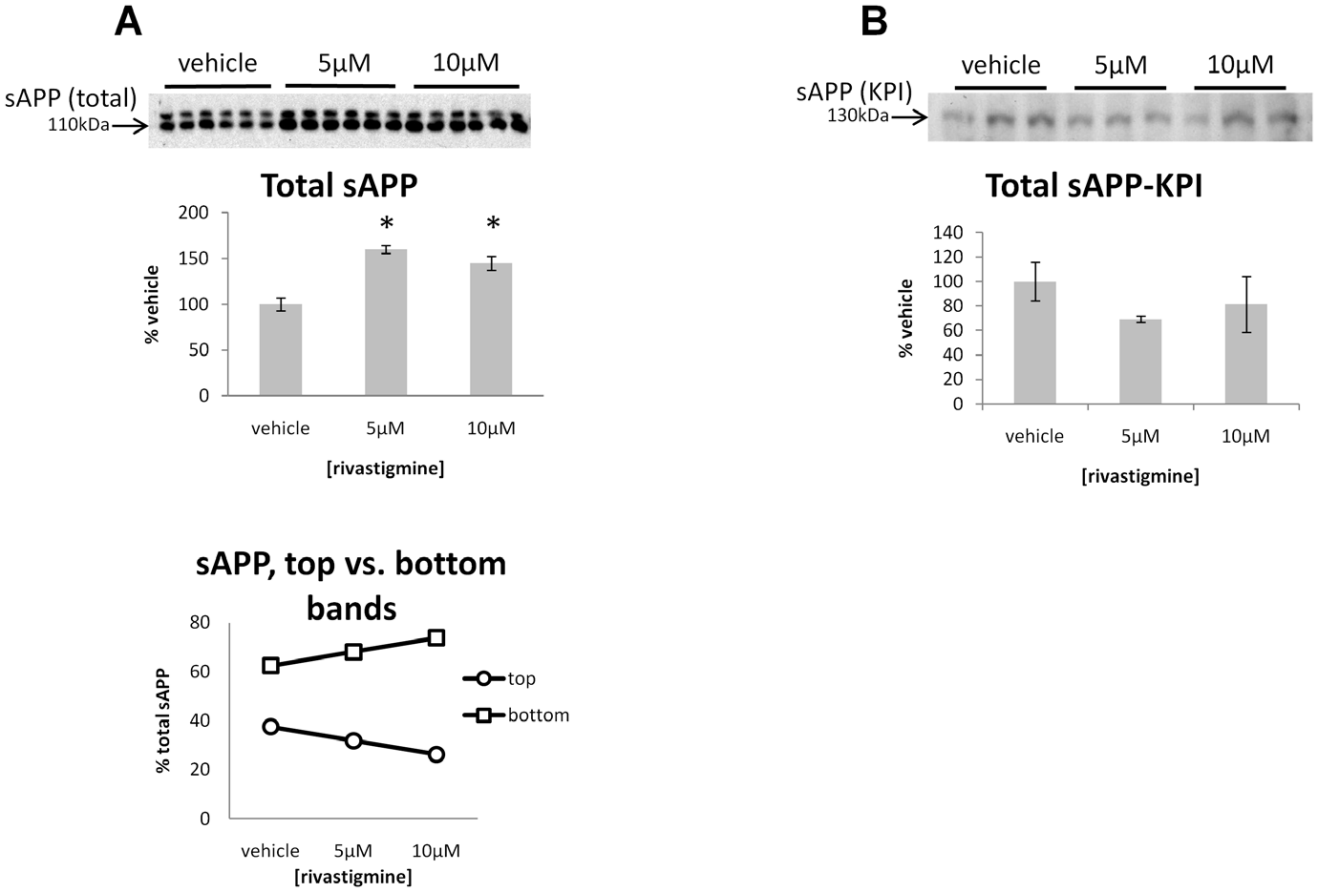
Rivastigmine increases the proportion of neuron-associated sAPP isoform to total secreted amyloid precursor protein (sAPP). **A**: Relative levels of total sAPP were measured by Western blot using the monoclonal 22C11 antibody. Densitometric quantification of the blots indicates that total sAPP is increased significantly at both rivastigmine concentrations tested (both p<0.05). The intensity of each band relative to the total sAPP was calculated and the top and bottom bands show diverging patterns, with the top band decreasing with rivastigmine treatment, and the bottom band increasing with rivastigmine treatment relative to total sAPP. **B**: Levels of the longer KPI-containing sAPP isoform were determined by Western blot using a KPI-specific antibody. A single band was detected in the media samples of these cells, and the molecular weight of this band coincides with the higher molecular weight band detected with the 22C11 antibody. Thus, the distinguishing characteristic between the top and bottom bands observed with 22C11 are differences in splicing. Levels of KPI containing APP are not altered by rivastigmine treatment.

This model of degenerating primary neurons was then applied to examine the preservative effect of rivastigmine on neuron structure. Cultures were treated with the drug at day 12 and treatment continued up to day 20 (figure 2A). The timing of this treatment was designed to determine the effect of rivastigmine during the period of neurodegeneration and glial proliferation observed previously. Notably, rivastigmine treatment preserved substantially, but not completely, neuronal structure during this period. Thus, rivastigmine treatment protected neurons from degeneration. In a separate experiment, sAPP was measured in the conditioned media of cells treated with vehicle or rivastigmine during the degenerating phase of the cell culture (figure 2B). Relative to day 12, the lower band was markedly reduced in vehicle treated cultures, while in rivastigmine treated cultures, there were significantly greater levels of low molecular weight sAPP. These results are consistent with a primarily neuronal source for the low molecular weight sAPP band and a glial source for the high molecular weight band as shown (figure 1). We further determined that the difference in apparent molecular weight represented by these two sAPP bands is the result of alternative splicing (see figure 4, below).

### Rivastigmine treatment increased the secretion of neuronal-associated sAPP isoform

Western blot data show a statistically significant increase in total sAPP secretion at 5 µM or 10 µM rivastigmine treatments, relative to vehicle (figure 4A; p<0.05). APP secreted from these cells typically formed a major doublet of bands on Western blots. The two major sAPP bands detected by the 22C11 antibody were quantified individually and are presented as percent of the total sAPP. Plotted this way, it is clear that the relative contribution of these two bands to the total sAPP is divergent, in that the bottom band increases and the top band decreases relative to the total with rivastigmine treatment. We have observed that the top band corresponds to longer splice variants of APP and tends to co-vary with glial cell density, whereas the lower molecular weight band, corresponding to shorter splice variants of sAPP, co-varies with neuronal cell density (figures 1 and 2). This shift toward lower molecular weight forms of sAPP suggests a stronger neuronal contribution to sAPP secretion. The ratio between the two sAPP bands was significantly shifted toward the low molecular weight form by both 5 µM (p<0.05) and 10 µM (p<0.01) rivastigmine treatment. This robust effect of rivastigmine on neuronal associated sAPP parallels cellular metabolic activity in neurodegenerating primary fetal rat cortical cultures (figure 3).

### Rivastigmine treatment had no effect on the level of KPI-containing secreted APP

Alternate splicing of sAPP was assessed by Western blot using an antibody specific to the KPI domain of the longer 751 and 770 amino acid APP isoforms (figure 4B). These data show that the higher molecular weight (∼130 kDa) of the two sAPP bands detected by the 22C11 antibody contains the KPI domain. Conversely, the lower (∼110 kDa) molecular weight band was not observed using the KPI-specific antibody. No difference in the levels of KPI containing APP was observed between vehicle and either concentration of rivastigmine. Combined with the cell type marker data (figure 1), these data suggest that the increase in total sAPP levels observed using the 22C11 antibody is produced principally by the neurons in these cultures.

### Rivastigmine shifts APP processing toward the α-secretase pathway

**Figure 5:**
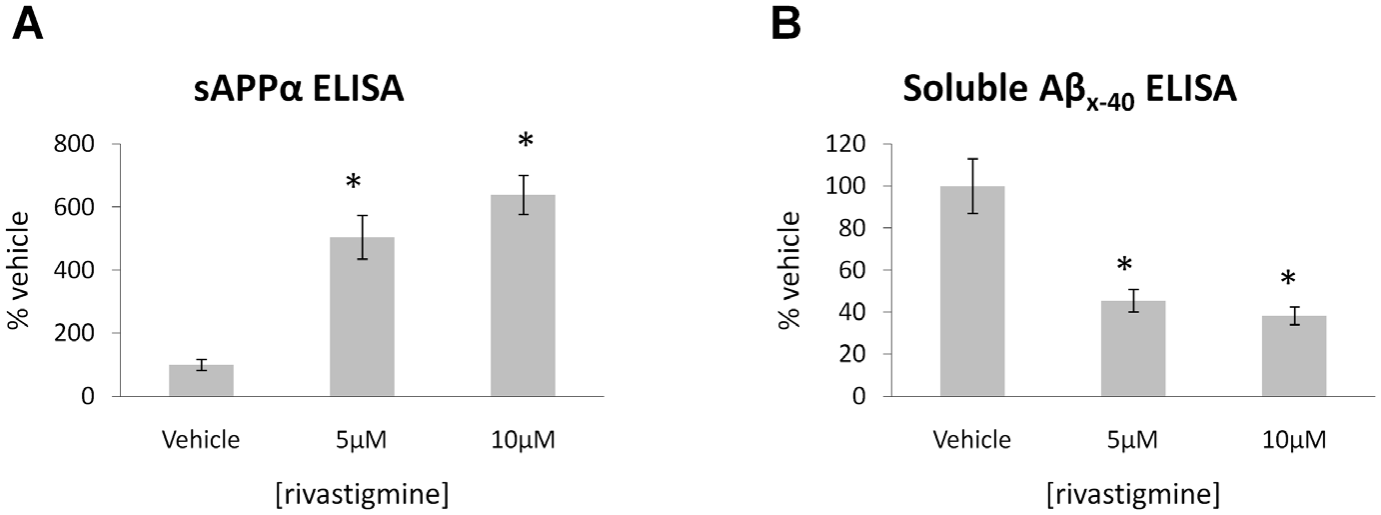
Rivastigmine shifts APP processing toward the α-secretase pathwayI. Levels of sAPPα and Aβ_x-40_ were measured in media samples of cultures treated with rivastigmine using ELISA techniques. A: sAPPα increased significantly in response to both 5 µM and 10 µM rivastigmine (both p<0.01), but the treatments were not significantly different rom each other suggesting a maximum effect of rivastigmine at or below 5 µM. B: Aβ_x-40_ levels were decreased by both concentrations of rivastigmine (both p<0.01). This effect was also not dose-dependent, suggesting a maximum effect at 5 µM or lower concentrations. Together, these data suggest a shift in the relative activities of the α- and β-secretase pathways, favoring production of the neurotrophic α-secretase product sAPPα while simultaneously reducing the neurotoxic β-secretase product Aβ.

ELISA analysis of media samples of rivastigmine-treated cultures revealed a significant increase in sAPPα in both 5 µM and 10 µM rivastigmine treatments (figure 5a; both p<0.01). In the same samples, ELISA analysis also showed that Aβ_x-40_ was significantly reduced (Figure 5b; both p<0.01). Together, these results suggest that rivastigmine alters the activities of the α- and β-secretase pathways in favor of sAPPα production. In both assays, the 5 µM and 10 µM treatments do not differ significantly from each other, suggesting a ceiling effect for these changes may occur below 5 µM rivastigmine. Measurement of Aβ_x-42_ was also attempted, but this was found to be below the limit of detection.

### Rivastigmine treatment had no effect on levels of APP holoprotein

**Figure 6:**
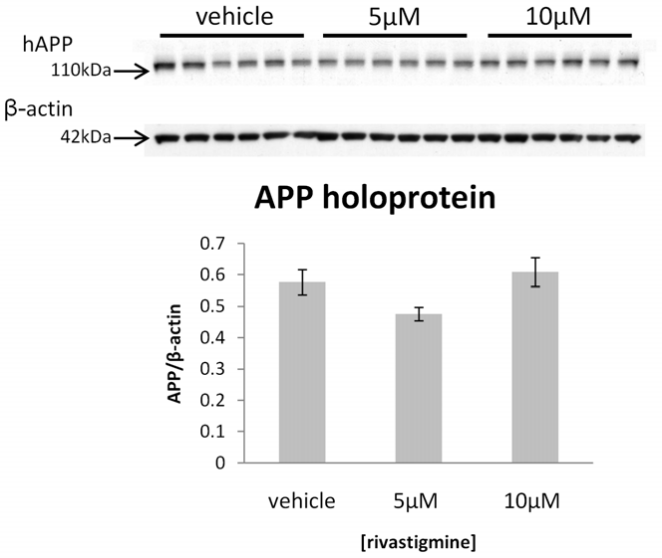
Rivastigmine does not alter APP holoprotein. APP levels in lysates of cerebrocortical cultures treated with vehicle or rivastigmine were measured by Western blot using the 22C11 antibody. No difference in APP holoprotein was found in either the 5 µM or 10 µM rivastigmine treatments, indicating that changes in sAPP levels are not the result of changes in total APP production in these cells.

To investigate effect of rivastigmine on APP gene expression, the uncleaved APP holoprotein (hAPP) was measured in the lysates of rivastigmine treated cells vs. vehicle treated control cultures. A predominant single band corresponding to intracellular hAPP was observed in the lysates, and there was no change in hAPP observed as a result of rivastigmine treatment as compared to vehicle (figure 6). These results suggest no change in total APP production in these cultures as a result of rivastigmine treatment, or perhaps a modest increase if APP holoprotein is combined with sAPP. A direct comparison cannot be made with the techniques applied here, though the potential effect of rivastigmine on total expression of APP may warrant further study.

### Rivastigmine-mediated increase in neuron-associated sAPP parallels elevations in synaptic protein markers

**Figure 7:**
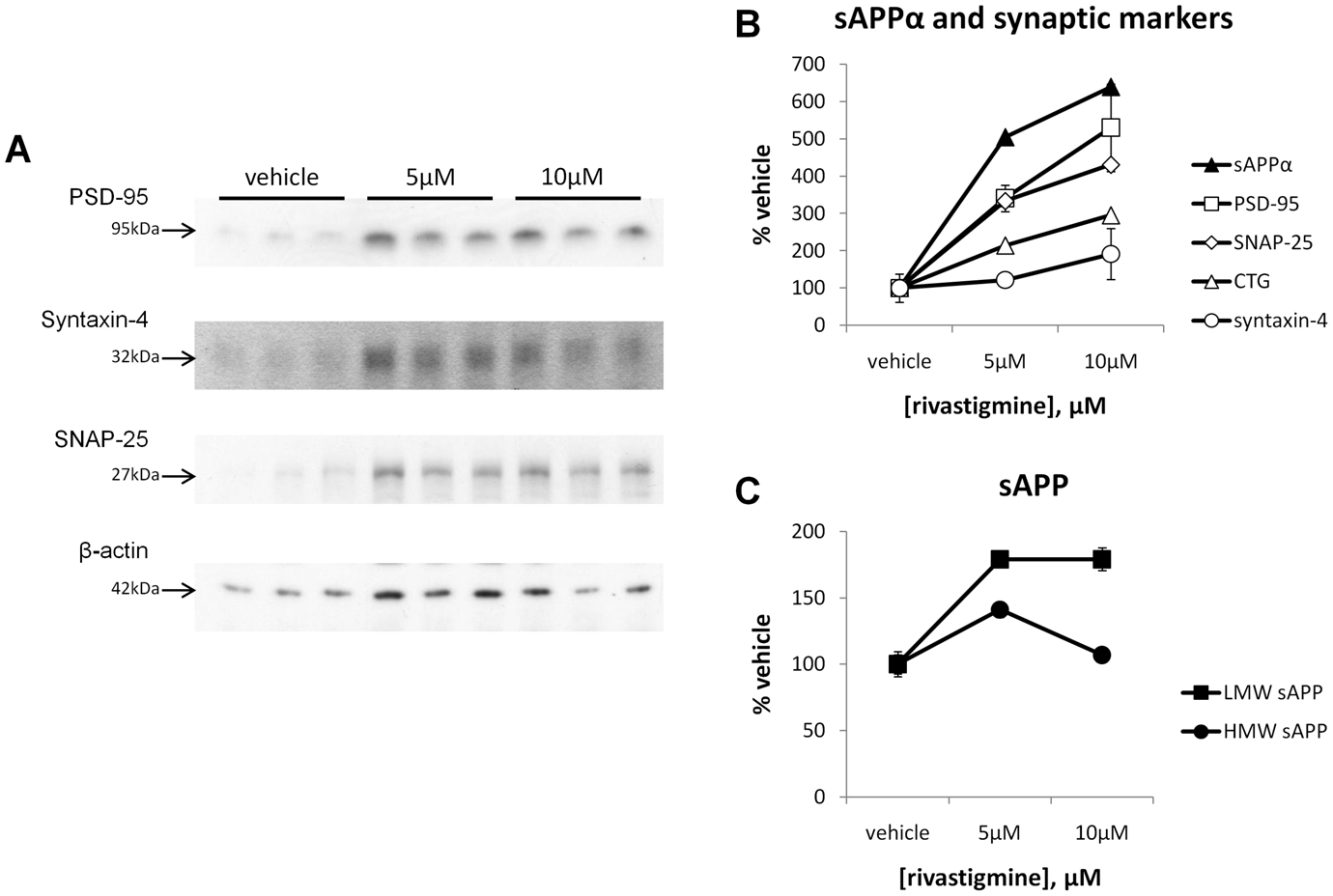
Rivastigmine-mediated increase in neuron-associated sAPP and sAPPα parallels increases in neuronal protein markers. Levels of low molecular weight neuronal sAPP (LMW-sAPP), high molecular weight glial sAPP (HMW-sAPP), and sAPPα were compared to levels of the presynaptic protein markers SNAP-25 and syntaxin-4, and the postsynaptic protein marker PSD-95. All values are expressed as a % of vehicle-treated cells for comparison. PSD-95 and SNAP-25 levels increased dose-dependently with rivastigmine treatment (both p<0.05), and syntaxin-4 levels increased but this change did not reach significance (7A and 7B). LMW-sAPP was increased with 5 µM rivastigmine but plateaued at the higher concentration. HMW-sAPP increased to a lesser extent at 5 µM, but was similar to vehicle at the higher concentration (7C). These data suggest that increased neuronal and decreased glial sAPP may be involved in the enhanced neuronal and synaptic marker stability that results from rivastigmine treatment, and that modulation of α-secretase may be involved in these effects.

In neurodegenerating primary fetal rat cerebrocortical cultures, significant increases in the pre-synaptic marker SNAP-25 and the post-synaptic marker PSD-95 were observed with 10 µM rivastigmine treatment (both p<0.05). A second pre-synaptic marker, syntaxin-4 was also increased by about two-fold compared to vehicle, but this effect did not reach significance (figure 7A and 7B). sAPPα (reproduced from figure 5 for comparison) increased dramatically with rivastigmine treatment, which follows the increasing patterns of synaptic markers and cell viability (figure 7B). Low molecular weight sAPP was increased significantly (p<0.05) by both concentrations of rivastigmine, while a smaller, but statistically significant, increase in high molecular weight sAPP was observed at 5 µM, but not 10 µM rivastigmine (figure 7C). These data suggest a differential involvement of these sAPP isoforms in the response to rivastigmine and synapse preservation.

### Rivastimine treatment increased levels of secreted APP in rat CSF samples

**Figure 8:**
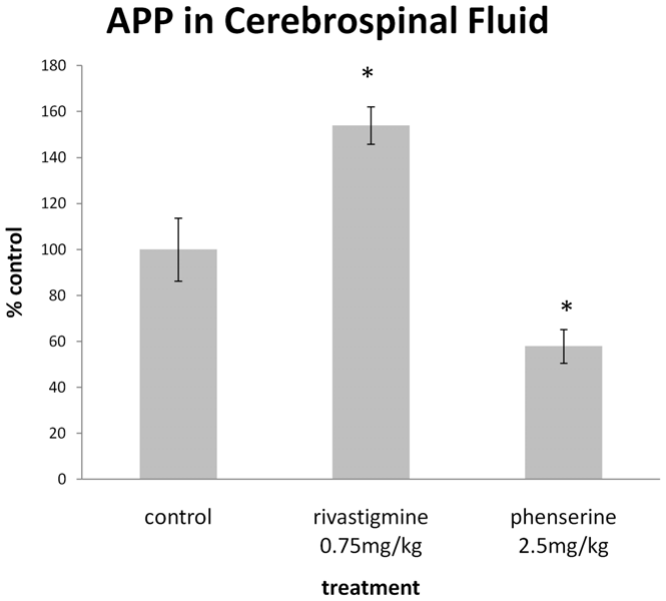
Rivastigmine elevates CSF sAPP levels in rodents. Separate groups of Fischer-344 male rats (n = 8–12) were administered saline (control) or rivastigmine (0.75 mg/kg) i.p. once daily for 21 days, and CSF was collected within 120 min of the final dose for quantification of sAPP by Western blot analysis probing with anti-APP antibody, 22C11. The APP synthesis inhibitor, phenserine (2.5 mg/kg, i.p. for 21 days) was administered to a concurrent group of animals as a positive control (*<0.05 vs control, Dunnett's t-test).

To elucidate whether drug-induced changes in APP levels determined in tissue culture translated to animals, male Fischer-344 rats were separated into groups of 8–12 animals and treated for 21 days with either rivastigmine (0.75 mg/kg i.p., prepared in 1 ml/kg volume isotonic saline) or vehicle (isotonic saline only) as described in ‘Materials & Methods’. All groups of treated rats gained weight in a manner similar to controls (data not shown), and there were no signs of drug-related toxicity. The presence of secreted total sAPP was detected in rat CSF samples by Western blot analysis. Densitometeric analysis revealed that rivastigmine (0.75 mg/kg for 21 days) significantly elevated CSF levels of sAPP to 154%±8.1 of saline controls (100%±13,7), whereas the positive control, phenserine (2.5 mg/kg for 21 days), reduced sAPP levels to 57.9%±7.4 (p<0.05 vs. control for both treatments; figure 8).

## Discussion

One major current goal for AD treatment is to restrict the progression and/or delay the onset of the disease. The currently available therapies based on ChEI or NMDA receptor antagonist treatments have failed to adequately attain this goal. Thus, a variety of alternate strategies are being pursued [11], including microRNA based mechanisms [39] and modulation of inflammatory processes [12], [40]. Secretion of APP through secretase-mediated cleavage has been a major focus of AD research due to the numerous possible roles of sAPP and the potentially toxic effects of the Aβ peptide. In fact, secreted products of APP and Aβ have been extensively studied to understand the molecular pathogenesis of AD as well as to target them for the development of suitable therapeutic agents. Indeed, a small, orally available naturally occurring compound, scillo-inositol, has been shown to penetrate into the brain and to rescue the memory impairment produced by soluble Aβ oligomers in animal models through a mechanism that restores hippocampal synaptic plasticity [41]. This promising compound is currently under investigation in early-stage clinical trials, but will require several years of additional investigations if it is to become widely available.

With regard to APP, it has been demonstrated previously that certain cholinesterase inhibitors can modulate APP secretion both in cell cultures and in vivo [42] . For example, the ChEI tacrine regulates sAPP levels in a variety of neuronal, glial and other cell types, but another ChEI, physostigmine, does not [43]–[45]. In experiments comparing the ChEIs physostigmine and phenserine, which are very similar in structure and potency for cholinesterase inhibition, differing effects on APP processing were observed [46]. This again suggests that changes in APP processing may involve mechanisms beyond those mediated by the cholinergic system. While the exact mechanism of action for most ChEIs are unclear, data suggesting mechanisms of action for certain ChEIs are available [47]. Therefore, this class of drugs represents a potential means, if appropriately optimized and translated, for modifying the course of AD by mitigating the downstream effects of Aβ, such as synapse loss. To this end, we have characterized the effects of the dual acetyl- and butyrylcholinesterase inhibitor, rivastigmine, on synaptic markers and APP cleavage in degenerating primary neuronal cultures.

We initially investigated the nature of the two most prominent forms of APP secreted from these neuronal cultures. Previous work has suggested that there is a time-dependent increase in neuronal markers, followed by their decline, which is concomitant with increased abundance of glial protein markers. Thus, we used the duration of the primary cell culture to obtain samples with peak neuronal marker expression (which occurs around day 12) and longer duration cultures with predominant glial markers. We confirmed by Western blot that samples taken at day 12, as compared to day 20, were enriched in neuronal protein, whereas the longer duration cultures were enriched in glial protein. The relative neuronal and glial populations of this mixed culture were also confirmed by immunocytochemistry. Our results contrast somewhat with other reported data using similar primary culture techniques. While our cultures begin to show glial activation and neurodegeneration around two weeks in vitro, others have reported viable neurons with limited glial activation for much longer periods. For example, using the same Neurobasal plus B27 media system, Brewer et al., observed healthy neurons and few activated glia at 4 weeks in culture [48]. The important differences between our technique and others that report longer-lived neuronal cultures include the use of mitotic inhibitors to prevent glial proliferation, use of neurons from later-stage embryonic animals, and refreshing only half the medium at a time. Our deviations from these alternative protocols induce time-dependent glial activation that results in neurodegeneration that is accompanied by a shift in the APP produced in these cultures.

These results are in agreement with previous observations that the KPI-containing APP is preferentially produced by cultured astrocytes, and only produced at much lower levels in primary neuronal cultures depeleted of glial cells [49]. Our results further demonstrate that this preferential expression of APP splice variants is maintained in cultures of mixed glial and neuronal cells. This lends additional interest to this cell culture model, given that a similar shift in APP isoforms, away from APP695 towards KPI-containing APP (i.e., from lower to higher moleculear weight forms of APP) has been observed in the cortices of AD-affected brains. Such a shift was also accompanied by increased levels of GFAP and Aβ, which suggests that glial activation and changes in APP isoforms are upstream events leading to AD pathology [50], and is partially recapitulated in this model. This shift in APP isoforms can be induced in animals by excitotoxic damage, and prevented by neuroprotective agents [51], suggesting that reversal of the shift from APP695 to KPI-containing APP may be an important indicator of neuroprotection. In the cell lysates, total levels of hAPP were not affected by rivastigmine treatment, thus it appears that the effect of rivastigmine on sAPP is related to altered processing rather than increased synthesis of APP. These findings are interesting both with respect to the effect of rivastigmine, and to future applications of this primary cell culture model. Rivastigmine treatment increased the neuron-related low molecular weight form of sAPP. This is suggestive of a mechanism by which rivastigmine may protect neurons by enhancing sAPP production, which may protect neurons from neurite retraction [33] and apoptosis [30]. And, with regard to the culture model, relative levels of these two major forms of sAPP could be used as indicators of neuronal viability in a variety of situations, for example, testing potential therapeutic agents.

We also investigated the relative activities of the α- and β-secretase mediated pathways by observing the APP processing products released into the medium by these cultured cells. We observed a simultaneous increase in the α-secretase product sAPPα, and a decrease in the β-secretase product Aβ. Given the neurotoxic nature of Aβ [26], [27] and the neurotrophic actions of sAPPα [52], this shift in the relative activities of the secretase pathways represents a likely mechanism for the neuroprotective effects of rivastigmine. These effects on Aβ and sAPP are not observed by all cholinesterase inhibiting drugs [42], [45], whiceh raises the possibility of non-cholinergic effects of rivastigmine in addition to the established acetylcholine-mediated actions. Further elucidation of alternate mechanisms of this and other drugs may provide new targets based on which better therapeutic agents can be developed.

Efforts are currently underway to combine the therapeutic potential of rivastigmine with that of the neuroprotective monoamine oxidase-B (MAO-B) inhibitor rasagiline. The addition of a carbamate group to rasagiline produced a molecule, designated TV3326, that is a structural hybrid of rivastigmine and rasagiline, and possesses both MAO-B and cholinesterase inhibiting properties [53]. This compound may act, at least in part, through activation of the PKC pathway [54]. Interestingly, the compound TV3279, which is a stereoisomer of TB3326 which does not inhibit MAO-B, has proven to be equally effective as the “active” compound in several paradigms. For example, both stereoisomers provided similar stimulation of the neurotrophic α-secretase pathway [54] and both provided greater protection from oxygen-glucose deprivation than rivastigmine [55]. These studies indicate that further mechanistic study and refinement of rivastigmine, and perhaps other neuroprotective agents, could be a fruitful strategy for producing the next generation of therapeutics to treat AD.

We report here that rivastigmine treated primary cultures have increased metabolic activity relative to untreated cells. We have also recently reported, and confirmed here, that presynaptic markers and neurite extension are increased by rivastigmine treatment [31]. Additionally, we show that rivastigmine also preserved the post-synaptic marker PSD95 in degenerating neurons. This hints that not only is the axonal exocytotic machinery preserved in rivastigmine-treated neurons, but so is the dendritic post-synaptic component of the neuron. We also measured levels of the vesicular protein syntaxin-4, and found an approximately two-fold increase with rivastigmine treatment, although this was much smaller than the increases in PSD-95 and SNAP-25, and did not reach statistical significance. In addition to neurons, syntaxin-4 was observed to be present at some level in astrocytic cells while SNAP-25 was not [56], which may explain the relatively higher levels of this protein in untreated cultures as compared to the other synaptic markers measured. Reductions in both pre- and post-synaptic proteins have been observed in AD and these losses correlate strongly with cognitive decline [57], [58]. Thus, a drug that can restore these synaptic markers may be useful in the treatment of AD. To this end, further study of these effects of rivastigmine are warranted, especially with respect to the shift toward α-secretase processing APP observed here, and its potential role in synaptic and neuronal preservation [52].

There are also interesting parallels between our cell viability results and published *in vivo* data collected in AD patients treated with rivastigmine. It was observed using positron emission tomography (PET) techniques that cerebral glucose metabolism declined in untreated AD patients, whereas glucose metabolism in rivastigmine-treated subjects was stabilized. These differences also correlated with cognitive performance [59]. In addition using PET techniques, nicotinic binding was observed to increase in rivastigmine treated subjects, which may represent a remodeling of cholinergic networks [60]. These similarities support the use of this kind of degenerating primary culture model to investigate AD drugs and drug candidates *in vitro*.

To assess whether the actions of rivastigmine in cell culture translated to animals, rivastigmine was administered to rats at a dose previously reported to induce brain cholinesterase inhibition [61], improve cognitive performance [62] and ameliorate inflammation without toxicity [63] . As assessed in CSF, rivastigmine significantly elevated sAPP levels by 154% compared to control. This contrasted with phenserine, likewise a carbamate type anticholinesterase, that has been reported to lower the rate of synthesis of APP in the brain. This compound was used as a positive control, which reduced CSF sAPP levels by 42.1%. This increase in sAPP in animals suggests the hypothesis that similar APP modulating and neuroprotective effects reported here may also occur in rivastigmine-treated animals. Brain hypometabolism has been established in AD, both as a correlate of reduced cognitive function [64] and a predictor of cognitive decline in the transition from mild cognitive impairment to AD [65]. Albeit that definitive diagnosis of AD is dependent on the presence of amyloid plaques and NFTs, synapse loss and neuronal atrophy, especially in the medial temporal lobes, correlates well with cognitive decline [28]. It is tempting to speculate that the hypometabolism detectable by PET is linked directly to Aβ-induced synapse loss in the AD brain, but this remains to be shown. To the extent that the action of rivastigmine *in vivo* is mirrored in this cell culture model, these results have interesting implications, both with respect to pharmacological treatment of AD, but also APP metabolism and function.

In summary, our goal has been to study the effect of rivastigmine on neuropreservation using a novel neurodegenerating primary rat cerebrocortical culture system [31]. The present work assumes significance as this demonstrates that specific sAPP isoforms, distinguishable by molecular weight, can be related to neuronal or glial sources, and that rivastigmine treatment enhances neuronal sAPP in degenerating neuronal cultures. This effect mirrors the trend of neuronal and synaptic proteins, and cellular viability. In addition, rivastigmine's effect on total sAPP levels was translated to *in vivo* studies in a wild-type animal strain. In these rats, sAPP increased similarly to the *in vitro* model, suggesting that the mechanism(s) of rivastigmine *in vivo* may also involve increasing sAPP release. Additionally, the cholinesterase inhibitor phenserine was shown to reduce sAPP in the CSF, which suggests that different cholinesterase inhibitors act through different mechanisms, which may include activities besides their cholinesterase enzyme inhibiting activities.

The data presented here suggest that changes in metabolic activity that result from rivastigmine treatment are associated with increased neuronal survival, which is accompanied by changes in the relative levels of the predominant isoforms of sAPP, and a shift in the relative activities of the α- and β-secretase pathways. While these data provide some insight into the mechanisms via which rivastigmine acts on neurons, further experimentation should be aimed at determining the effects of rivastigmine treatment on neurons in order to guide future drug development efforts.
